# Galectin-3—A New Player of Kidney Damage or an Innocent Bystander in Children with a Single Kidney?

**DOI:** 10.3390/jcm10092012

**Published:** 2021-05-08

**Authors:** Eryk Latoch, Katarzyna Konończuk, Anna Jander, Elżbieta Trembecka-Dubel, Anna Wasilewska, Katarzyna Taranta-Janusz

**Affiliations:** 1 Department of Pediatric Oncology and Hematology, Medical University of Bialystok, 15-274 Białystok, Poland; eryklatoch@gmail.com (E.L.); kononczukk@gmail.com (K.K.); 2 Department of Pediatrics, Immunology and Nephrology, Polish Mother’s Memorial Hospital Research Institute, 93-338 Łódź, Poland; ajander@wp.pl; 3 Department of Pediatrics, Faculty of Medical Sciences in Zabrze, Medical University of Silesia in Katowice, 41-800 Zabrze, Poland; etdubel@interia.pl; 4 Department of Pediatrics and Nephrology, Medical University of Bialystok, 15-274 Białystok, Poland; annwasil@interia.pl

**Keywords:** children, chronic kidney disease, cystatin C, galectin-3, solitary functioning kidney

## Abstract

The aim of this study was to evaluate the galectin-3 (Gal-3) level in children with a congenital solitary functioning kidney (cSFK) and determine its association with common renal function parameters. The study consisted of 68 children (49 males) with cSFK. We demonstrated that children with cSFK had a lower level of galectin-3 than that of healthy subjects (*p* < 0.001). No significant differences in serum cystatin C (Cys C) levels between the cSFK children and the reference group were found. The subjects with cSFK and reduced estimated glomerular filtration rate (eGFR) had significantly higher levels of Gal-3 and Cys C compared to those with normal eGFR (*p* < 0.05). Children with eGFR <60 mL/min/1.73 m^2^ showed significant statistical differences between the values of area under ROC curve (AUC) for Gal-3 (AUC 0.91) and Cys C (AUC 0.96) compared to that for creatinine level (AUC 0.76). Similar analyses carried out among cSFK children with eGFR <90 mL/min/1.73 m^2^ revealed an AUC value of 0.69 for Gal-3, 0.74 for Cys C, and 0.64 for creatinine; however, no significant superiority was shown for any of them. The receiver operating characteristic (ROC) analyses for identifying the SFK children among all participants based on the serum levels of Gal-3 and Cys C did not show any diagnostic profile (AUCs for Gal-3 and Cys C were 0.22 and 0.59, respectively). A positive correlation between the Gal-3 and Cys C concentrations was found (*r* = 0.39, *p* = 0.001). We demonstrated for the first time that Gal-3 might play an important role in the subtle kidney damage in children with cSFK. However, further prospective studies are required to confirm the potential applicability of Gal-3 as an early biomarker for kidney injury and possible progression to CKD.

## 1. Introduction

A solitary functioning kidney (SFK) is a common abnormality in the spectrum of congenital anomalies of the kidney and urinary tract (CAKUT). It may cause chronic kidney disease (CKD) in approximately 50% of cases [1,2]. Children diagnosed with congenital SFK (cSFK) are at higher risk of kidney diseases and hypertension later in life. The slightly progressive deterioration of renal function over time is mainly due to reduced nephron endowment resulting in glomerular hyperfiltration and subsequent hyperperfusion injury of glomeruli [3,4]. However, in recent years, the influence of other factors in renal function decline in individuals with SFK has also been emphasized [5,6]. Currently, the available methods, including estimated glomerular filtration rate (eGFR), renal nuclear scans, or ultrasonography, do not allow for early detection of renal impairment, and these are not good predictors of the future course of kidney disease. Since the mechanisms that result in renal function decline in children born with SFK are only partly understood, there is a need for new biomarkers to distinguish those at higher risk.

Galectin-3 (Gal-3) is a β-galactoside-binding lectin protein coded by a single gene (LGALS3) located on chromosome 14. It is widely expressed in human tissues, including many types of cells, such as epithelial and endothelial cells; neurons; and many types of immune cells. The contribution of Gal-3 to various cellular functions depends on its locations (intra- or extra-cellular) and plays an important role in cell-to-matrix and cell-to-cell interactions [7]. Hence, it is involved in numerous physiological pathways, including embryogenesis, cell differentiation and proliferation, inflammation, fibrosis, and angio- and onco-genesis [8]. Interestingly, the expression of galectin-3 is substantially upregulated during embryogenesis and in the first years of development. It is more specific to particular organ tissues, such as kidney, bone, or liver, compared to adults [9]. Upregulation of Gal-3 in the ureteric bud and its derivations is crucial for the formation of ureteric bud branching [10,11,12]. Moreover, Henderson et al. demonstrated that Gal-3 expression was upregulated in a mouse model of progressive renal fibrosis in unilateral ureteric obstruction. The absence of Gal-3 protected against renal myofibroblast accumulation and activation in fibrosis [13]. Available experimental and clinical data support the hypothesis that Gal-3 may be both a biomarker and a biotarget. As a biomarker, increased or increasing Gal-3 may identify patients with excessive risk for poor outcomes. As a biotarget, possibly according to the galectin level, it would be plausible to estimate which patients could benefit from intensified therapy with Gal-3 inhibition [14]. Studies in humans have demonstrated that Gal-3 may be used as a useful diagnostic and prognostic biomarker in kidney diseases, cardiovascular diseases, and certain types of cancer [9].

To date, there is a limited number of studies on the role of Gal-3 in children with cSFK. Serum creatinine (cr.) is widely used to estimate glomerular filtration rate; however, due to its vulnerability to many factors, such as diet, lean mass, age, sex, and hydration status, it is a poor marker of early renal damage with many limitations. Moreover, a significant increase in creatinine level occurs long after the onset of deterioration of renal function. Cystatin C (Cys C) plays important pleiotropic roles in, among other things, cellular protein catabolism and vascular pathophysiology, in particular, regulating cathepsins S and K. In recent years, it has been postulated that Cys C may be a better biomarker of kidney function compared to traditional diagnostic measures; however, there is still no widespread recommendations for its use [15,16,17,18]. In this regard, new biomarkers of early kidney damage are sought.

The aims of this multicenter preliminary study were to evaluate the galectin-3 level in children with a congenital SFK and to determine whether there is a relationship between the tested marker and commonly used methods of accessing renal function.

## 2. Materials and Methods

Sixty-eight children (49 males) with a congenital solitary functioning kidney were enrolled in the study. The participants were recruited from three Polish units (the Department of Pediatrics and Nephrology, Medical University of Bialystok; the Department of Pediatrics, Immunology and Nephrology, Polish Mother’s Memorial Hospital Research Institute, Łódź; and the Department of Pediatrics, Faculty of Medical Sciences in Zabrze, Medical University of Silesia in Katowice) between 2018 and 2020. The study’s inclusion criteria were congenital unilateral kidney agenesis diagnosed under the age of 18 and availability of clinical data. Since the levels of the proteins studied can be affected by many diseases, children with any comorbidities or current infection were excluded from the survey. Written informed consent to the release of medical record information was obtained from parents or guardians and children aged 16 or older. The study was conducted according to the guidelines of the Declaration of Helsinki and was approved by the Institutional Review Board of the Medical University of Bialystok (APK.002.73.2020). The reference group consisted of 20 healthy peers (14 males), who were children of the Department of Pediatrics and Nephrology employees. They were full-term, normal birth weight children, and they were not receiving any medication at the time of the study.

At the follow-up visit, all participants underwent a clinical examination and anthropometric measurements using standard techniques. Body mass index (BMI) was calculated using the following formula: weight (kg)/height^2^ (m^2^). Systolic (SBP) and diastolic (DBP) blood pressure were measured with a standardized sphygmomanometer. Hypertension was defined as a mean SBP and/or DBP level  ≥95th percentile adjusted for age, sex, and height (based on the mean of the three measurements with two-minute intervals). Glomerular filtration rate was assessed by the updated Schwartz formula (eGFR = 0.413 × (height in cm/serum creatinine in mg/dL)). Excretion of urinary albumin (albuminuria) was determined in the urine collected during a 24 h period. In younger children, due to the difficulty in collecting 24 h urine, the albumin/creatinine ratio in the morning urine sample (UACR) was assessed. Albuminuria was defined as a daily excretion in the range of 30–300 mg/24 h and UACR 30–300 mg/g creatinine. For all children, the clinical history was collected from the medical records or patient database. On ultrasound examination of the abdominal cavity, a solitary functioning kidney was identified and confirmed on dynamic renoscintigraphy.

The collected blood samples were stored frozen at −80 °C after a 12 h overnight fast. The serum creatinine level (cr.) was determined by the Jaffe reaction. The serum galectin-3 expressed as picograms per milliliter (pg/mL) was measured with a commercial immunoassay kit (Wuhan EIAab^®^ Science CO, Wuhan, Hubei, China) in accordance with instructions for the ELISA kit. The serum cystatin C level (expressed in milligrams/liter—mg/L) was determined by the nephelometric immunoassay (PENIA) method on the Dade Behring nephelometer systems (BNA, BN II).

According to Kidney Disease: Improving Global Outcomes (KDIGO) guidelines, CKD is diagnosed based on structural or functional impairment of renal function and/or reduction of GFR to less than 60 mL/min/1.73 m^2^, lasting more than three months [19].

Statistical analyses were performed using the STATA 12.1 version (StataCorp, College Station, TX, USA). Variable distribution was tested with the Shapiro–Wilk tests. The normally distributed data were presented as mean ± SD and the skewed data as the median and interquartile range (IQR). After the examination of distribution and skew, correlations were assessed using Spearman’s rank correlation for non-parametric data and Pearson’s correlation for parametric data. In the analysis of the categorical variables, the chi-square test or Fisher exact test was used. The *t*-Student test or Mann–Whitney U test was used to compare the continuous variables. Univariable and multivariable logistic regression analyses between the galectin-3 and cystatin C levels and reduced eGFR (<90 mL/min/1.73 m^2^) were performed. The receiver operating characteristic (ROC) curve was used to determine the diagnostic value of the examined markers and the optimum cut-off values. The statistical significance was determined at 0.05.

## 3. Results

The demographic characteristics of the study and the reference group are presented in Table 1. The median time at the study was 7.26 years (range: 3 months–18.1 years). Age, gender, and anthropometric measurements did not differ between the children with a cSFK and the reference group. In the study group, cSFK on the left side predominated, and boys outnumbered the girls. Six (8.8%) hypertensive children in the cSFK group were identified.

**Figure 1:**
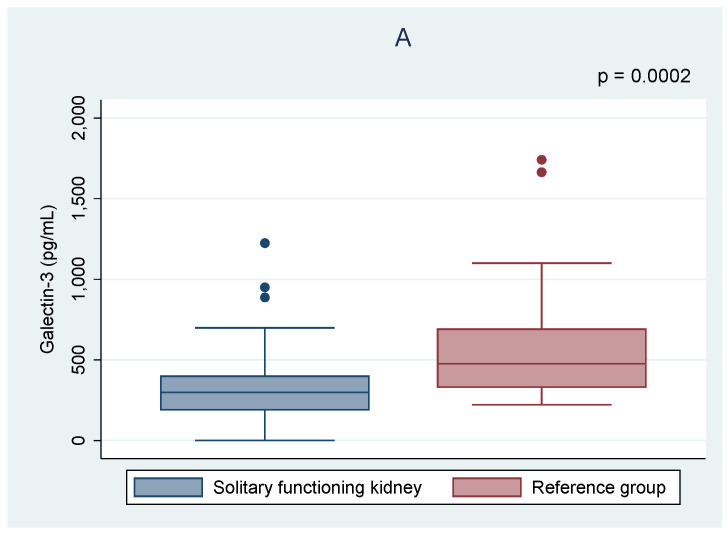

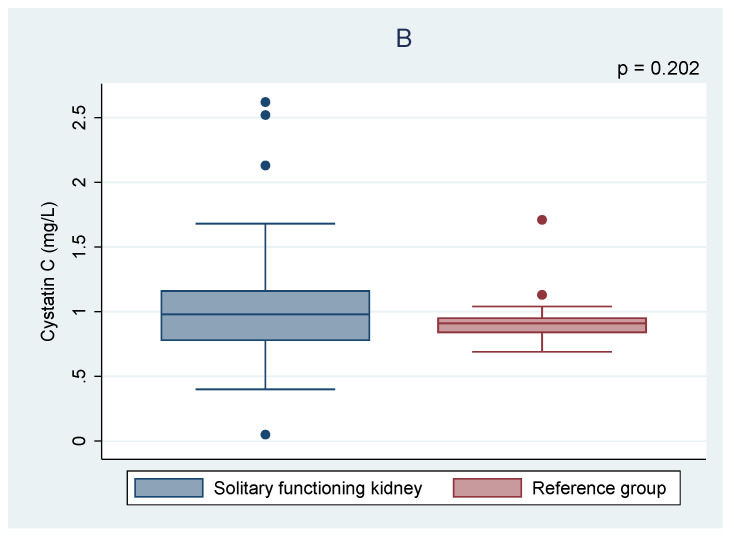
Comparison of the galectin-3 (**A**) and cystatin C (**B**) levels between the children with solitary functioning kidney and the reference group.

As shown in Table 2, there were no significant differences in the serum creatinine level and eGFR between the analyzed groups. Higher levels of serum urea and uric acid were found in the cSFK children. Only galectin-3 differed significantly between the groups, and its lower concentrations were observed in cSFK patients compared to healthy controls (302.8 vs. 475.9 pg/mL, *p* < 0.001) (Figure 1). Five of all children presented albuminuria (7%). There was no correlation between albuminuria and galectin-3 level among children with cSFK (*p* < 0.05).

**Figure 2:**
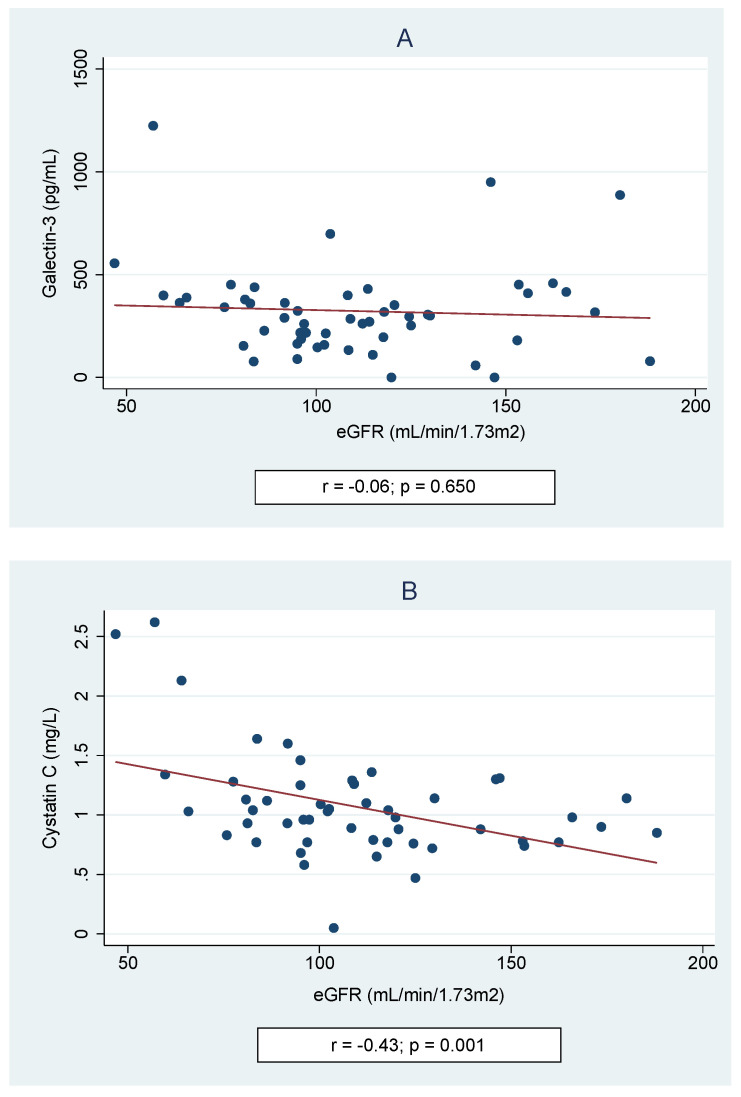
Spearman correlations of galectin-3 (**A**) and cystatin C (**B**) according to estimated glomerular filtration rate (eGFR).

Of all children with cSFK, 13 (19%) had a decreased eGFR—10 (14.7%) in the range of 90 and 60 mL/min/1.73 m^2^ and 3 (4.4%) between 59 and 30 mL/min/1.73 m^2^. As shown in Table 3, the mean levels of both Gal-3 and Cys C were significantly higher in the subset of patients with a reduced eGFR compared to those cSFK patients who had a normal eGFR (412.1 ± 275.0 vs. 293.3 ± 202.3 pg/mL, *p* = 0.049 and 1.41 ± 0.62 vs. 0.95 ± 0.29, *p* = 0.012, respectively). In contrast, the Spearman correlations showed no correlation between eGFR and Gal-3 level (*r* = −0.06, *p* = 0.65), whereas a negative correlation between Cys C and eGFR was observed (*r* = −0.43, *p* = 0.001)—Figure 2. Moreover, the higher odds ratio of a reduced eGFR (<90 mL/min/1.73 m^2^) among cSFK children and a high cystatin C level was observed (OR 15.29, *p* = 0.012).

**Figure 3:**
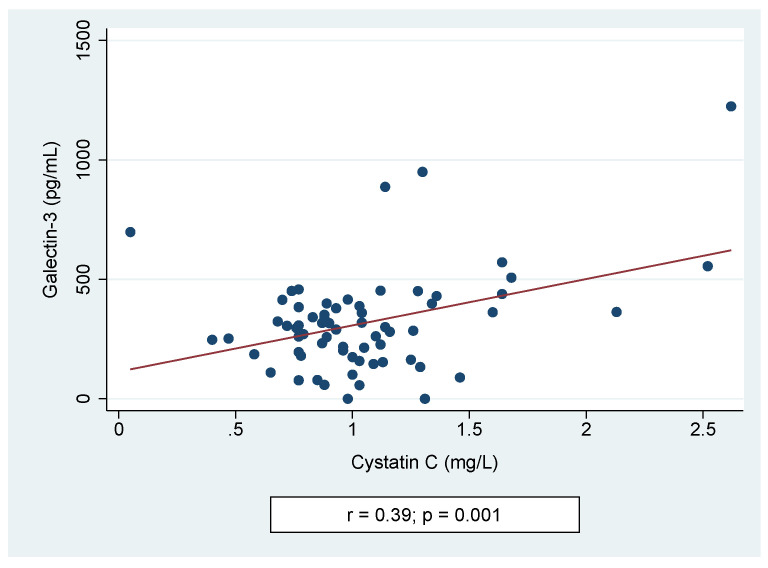
Spearman correlation of galectin-3 and cystatin C levels.

Univariable linear regression analysis showed significant correlations between the levels of Gal-3 and age at study (coefficient (coeff.) −15.9, *p* = 0.002). Other independent variables, such as BMI *Z*-score (coeff. 25.45, *p* = 0.261), SBP (coeff. 0.41, *p* = 0.771), DBP (coeff. 1.41, *p* = 0.451), eGFR (coeff. −0.44, *p* = 0.654), serum levels of urea (coeff. −7.15, *p* = 0.001), or uric acid (coeff. −62.6, *p* = 0.289), did not significantly affect the Gal-3 concentration. Univariate analysis between the cystatin C level and potentially confounding factors showed an association with eGFR (coeff. −0.006, *p* = 0.001), but not with BMI *Z*-score (coeff. 0.024, *p* = 0.604), SBP (coeff. −0.002, *p* = 0.615), DBP (coeff. −0.004, *p* = 0.434), serum levels of urea (coeff. 0.54, *p* = 0.784), or uric acid (coeff. 0.13, *p* = 0.347). A positive correlation between the levels of Gal-3 and Cys C was found (*r* = 0.39, *p* = 0.001)—Figure 3. 

**Figure 4:**
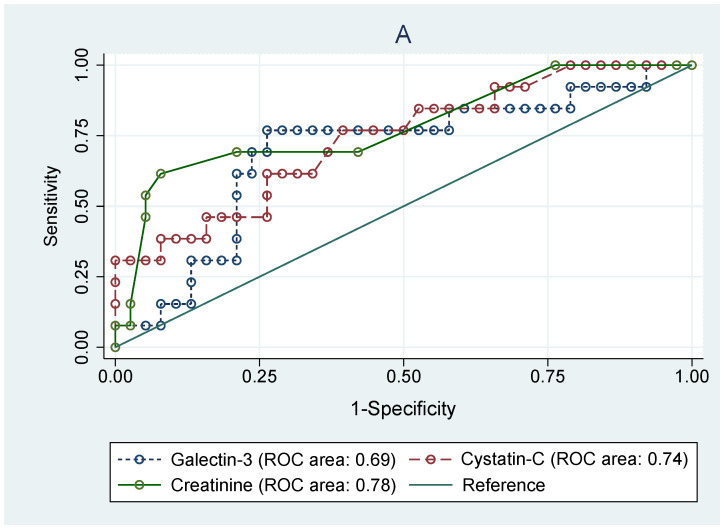

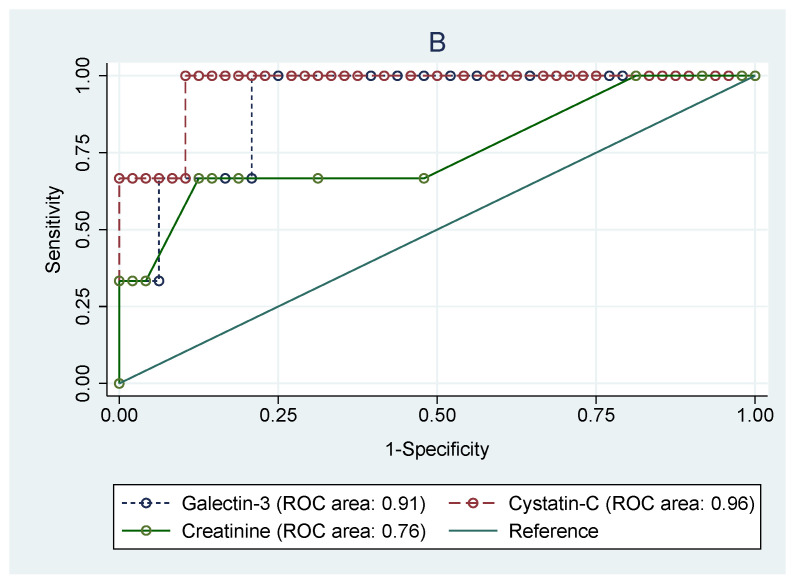
Receiver operating characteristic (ROC) analyses for prediction of decreased eGFR below 90 mL/min/m^2^ (**A**) and below 60 mL/min/m^2^ (**B**) based on the serum levels of galectin-3, cystatin C, and creatinine in congenital solitary functioning kidney children.

As shown in Figure 4, receiving operating curve analyses in cSFK subjects were conducted in order to define the diagnostic profile of Gal-3 level in identifying children with decreased eGFR (Figure 4A) below 90 mL/min/1.73 m^2^ and (Figure 4B) below 60 mL/min/1.73 m^2^) compared to the Cys C and creatinine levels. In the first subset of patients (Figure 4A), the AUC for the Gal-3 level was 0.69 with the best cut-off value of 227.2 pg/mL (sensitivity: 84.6%; specificity: 41.1%), whilst Cys C and cr. showed a diagnostic profile describing the AUC of 0.74 and 0.78, respectively (*p* = 0.643). The AUCs for the group with an eGFR below 60 mL/min/1.73 m^2^ were 0.91 for Gal-3, 0.96 for Cys C, and 0.76 for creatinine (*p* = 0.029). The ROC analyses for identifying the SFK children among all participants based on the serum levels of Gal-3 and Cys C were performed. However, they did not show any diagnostic profile (AUCs for Gal-3, Cys C, and creatinine were 0.22, 0.59, and 0.64, respectively). 

## 4. Discussion

In recent years, the number of patients with chronic kidney disease has increased due to many nephropathies. Currently, the greatest challenge is to distinguish individuals who may be at increased risk of CKD before the onset of overt disease. Data from the literature indicate that children with ipsilateral CAKUT had higher proportions of renal injury (48.3% versus 24.6%), while longitudinal models showed a decrease in glomerular filtration rate from the beginning of puberty onwards [20]. It was initially thought that children with SFK, despite compensatory renal hypertrophy, did not experience serious medical conditions. This hypothesis was supported, among others, by a study of adult kidney donors who lived for more than 20 years after donation. The authors showed no serious complications regarding impaired renal function, the incidence of hypertension, or proteinuria [21,22]. However, the studies in the subsequent years showed that kidney donors had an increased risk of end-stage renal diseases compared with matched healthy non-donors [23]. Moreover, the studies in patients with cSFK revealed that this particular population is at higher risk of hypertension, deterioration in kidney function, and proteinuria [24,25].

Due to the fact that current methods assessing kidney function do not reflect an early kidney injury, new biomarkers that distinguish children at an increased risk of kidney damage are needed. This necessity is also attributed to the fact that the mechanisms leading to nephron damage have not been fully elucidated. Some of the candidate biomarkers include galectin-3.

In this multicenter, preliminary study, we aimed to investigate a potential biomarker of kidney injury, galectin-3, in children with a congenital SFK and to determine its relationship with commonly used methods, the levels of creatinine, and eGFR.

To our best knowledge, this is the first clinical study of the association between Gal-3 and cSFK in children. We demonstrated herein that children with unilateral congenital agenesis had a significantly lower level of Gal-3 than that of a healthy reference group. The normal range of Gal-3 may differ in individuals with normal kidneys from those with cSFK. No data were found on the Gal-3 level according to the unilateral absence of kidney when reviewing the literature. However, Gal-3 is known to play an important role in nephrogenesis, and its concentration in different renal structures varies with the period of embryogenesis. In the mature kidneys, Gal-3 is only poorly found in the distal tubules and in a subset of collecting duct cells. On the other hand, during development, it is highly expressed in ureteric bud brunches of the metanephros [8,26]. Therefore, we cannot exclude the dependency of Gal-3 concentration on renal mass. The absence of galectin-3 might also be related to protection against renal fibrosis and consequently better renal outcomes among children with cSFK. Many types of cells expressed Gal-3 at different times, pointing to a specific role at different times. It has been demonstrated that the Gal-3 knockout mouse has a smaller number of glomeruli than the wild type with kidney hypertrophy. Lesser tissue damage was observed in the knockout mouse, which may reflect a reduced extent of renal injury [27]. Moreover, the univariable analysis showed a significant negative correlation between Gal-3 and age at study in children with cSFK, which suggests decreasing Gal-3 levels with age during childhood. However, conclusions from this analysis should be drawn with caution, as the level of Gal-3 may depend on many factors, and further longitudinal studies on a large cohort of participants need to be conducted. In our opinion, the presented data on decreased galectin-3 in SFK children could lead to new insights into the complex pathogenesis of SFK.

Interestingly, despite the fact that children with cSFK had a lower level of Gal-3 compared to controls in this study, further analysis of children with cSFK in relation to eGFR values (below and over 90 mL/min/1.73 m^2^ as well as below and over 60 mL/min/1.73 m^2^) showed that individuals with reduced eGFR had a significantly higher Gal-3 level compared to those who had normal eGFR. This finding suggests that Gal-3 may play an important role in the subtle kidney damage in children with SFK, which was also confirmed in previous studies [28,29,30,31,32]. There are many studies highlighting the role of an elevated level of Gal-3 in various kidney diseases, including acute kidney disease (AKI), CKD, diabetic nephropathy, cardiorenal syndrome, polycystic kidney diseases, renal cell carcinoma, and glomerulonephritis [8]. An elevated serum level of Gal-3 has been associated with a higher risk of CKD and renal dysfunction, suggesting that Gal-3 can predict renal damage years before CKD is detected clinically, facilitating targeted treatment and disease. To date, among many diseases, the relationship between Gal-3 and cardiovascular disease has been best understood. It is well known that patients with CKD are at a much higher risk of mortality due to cardiovascular disease, and these two conditions are strongly interrelated. Furthermore, Gal-3 inhibition is considered a therapeutic pathway to prevent cardiac inflammation and fibrosis [7,33]. Similar investigations of Gal-3 inhibition in animal models with experimental kidney diseases have been conducted to date [8].

There is a well-established linkage between the level of Cys C and renal damage. Therefore, we set out to additionally refer the results of Gal-3 to Cys C level. Our previous study demonstrated that increased serum of Cys C concentration in patients with unilateral cSFK occurs after 12 years of age and correlates with compensatory overgrowth of the kidney [34].

As previously mentioned, some authors emphasize that the use of a combination of different biomarkers may be beneficial in detecting kidney injury. Ji et al. showed that the joint analysis of Gal-3, Cys C, and creatinine may distinctly improve the diagnostic accuracy in CKD patients [32]. The present study has demonstrated a positive correlation between Gal-3 and Cys C as well as elevated levels of both biomarkers in individuals with reduced eGFR, which may suggest their common role in the pathomechanisms of renal tissue damage in children with cSFK.

Moreover, the ROC analyses carried out among cSFK children to identify subjects with eGFR < 90 mL/min/1.73 m^2^ revealed an AUC value of 0.69 for Gal-3, 0.74 for Cys C, and 0.64 for creatinine. However, no significant superiority was shown for any of them. Further analysis in the subset of participants with eGFR <60 mL/min/1.73 m^2^ showed significant statistical differences between AUC values for Gal-3 (AUC 0.91) and Cys C (AUC 0.96) compared to that of creatinine level (AUC 0.76). All of these findings may suggest a valuable role of Gal-3 in predicting renal impairment. Yet, it may be hazardous to draw definitive conclusions from the comparison of galectin-3 levels according to eGFR groups, as the galectin-3 level was decreased compared to healthy peers. We have to temper our conclusions, as this is just a preliminary study, and assessment of children with GFR <90 mL/min/1.73 m^2^ showed only an increasing tendency in comparison with the reference group, which requires confirmation in further studies on a larger group of patients. Similar analyses for identifying the cSFK children among all participants based on the serum levels of Gal-3, Cys C, and cr. did not show any diagnostic profile (AUCs were 0.22, 0.59, and 0.64, respectively). It seems that galectin-3 may be a useful marker for identifying individuals at high risk of developing new-onset CKD, and it could be a relevant target for pharmacotherapy for the prevention of CKD incidence and progression.

In our analysis, the prevalence of hypertension was 8.8%, while in previous studies in children with SFK, the number of hypertensive patients ranged from 13 to 26% [25,35]. In the KIMONO study by Westland et al., the authors concluded that an SFK from childhood implies a substantial risk for hypertension [20]. The relatively small number of hypertensive patients in our study may be due to the fact that most of the enrolled participants were prepubertal, as well as the small group size.

There are several limitations to this study. Firstly, the number of patients included in this study was small, which may influence the final results. Secondly, the cross-sectional study should be interpreted in a context of possible bias that may occur, and the diagnostic value of estimated markers can only be speculated upon. Finally, the study group consisted of children of different ages and pubertal status. There is still no unanimity among investigators regarding the best estimation of GFR in prepubertal children and which formula reliably reflects kidney function in this group of children. Another limitation of the present study is that GFR was only estimated from creatinine values instead of being measured by standard techniques, e.g., iohexol plasma clearance. However, this is a rapid, precise, and accurate measurement of kidney function essential for daily workup with children. The strengths of our study include a homogeneous group of children with unilateral congenital agenesis, the multi-center design, and the lack of ethnic diversity.

Pooling all data, we demonstrated that children with congenital SFK had a lower level of galectin-3 than that of healthy subjects. We provided new data that children with cSFK and reduced eGFR had significantly higher levels of Gal-3 compared to those with normal eGFR. Moreover, a positive correlation between Gal-3 and Cys C concentrations was found. All these findings suggest that Gal-3 may be a new player of the subtle kidney damage in children with cSFK. However, further prospective studies are required to confirm the potential applicability of Gal-3 as an early biomarker for kidney injury and progression of CKD. There are still several questions that need to be answered. Among other things, it has not yet been established whether age affects estimated biomarker concentrations. Another issue that needs clarification is the relationship between Gal-3 levels and renal mass.

Considering all of the above limitations of the study, we believe that these results have important diagnostic potential, which, however, requires further verification in prospective, longitudinal studies.

## Figures and Tables

**Table 1:** Clinical characteristics of patients with a solitary functioning kidney and the reference group.

	Congenital Solitary Functioning Kidney	Reference Group	*p*
Patients (*n*)	68 (100%)	20 (100%)	-
Male (*n*)	49 (72%)	14 (70%)	1.000
Female (*n*)	19 (28%)	6 (30%)	
Age at diagnosis (years)	0.08 (0.00–4.69)	-	-
Age at study (years)	7.26 (4.16–11.78)	5.1 (0.63–11.71)	0.120
Laterality (left/right)	45/23	-	-
BMI (Z-score)	0.35 (−0.41–0.96)	−0.91 (−1.15–1.13)	0.427
SBP (mmHg)	109 (100–117)	99 (94–110)	0.241
DBP (mmHg)	65 (60–73.5)	65 (56–68)	0.540

BMI, body mass index; SBP, systolic blood pressure; DBP, diastolic blood pressure. Data are presented as median and interquartile range; categorical variables are presented as numbers (%).

**Table 2:** Summary of the biochemical parameters in children with a congenital solitary functioning kidney and the reference group.

	Congenital Solitary Functioning Kidney	Reference Group	*p*
Total	68	20	
Galectin-3 (pg/mL)	302.8 (200.7–401.6)	475.9 (329.8–700.0)	<0.001
Cystatin C (mg/L)	0.98 (0.79–1.18)	0.91 (0.83–0.95)	0.202
Urea (mg/dL)	28.0 (21.9–33.5)	19.0 (16.5–23.2)	0.011
Uric acid (mg/dL)	4.7 (4.0–5.3)	3.35 (3.1–4.1)	0.014
Serum creatinine (mg/dL)	0.4 (0.4–0.6)	0.4 (0.3–0.5)	0.070
eGFR (mL/min/1.73 m^2^)	106.0 (87.6–128.3)	112.6 (102.9–131.4)	0.583

eGFR, estimated glomerular filtration rate. Data are given as the median (Me) with interquartile range (IQR).

**Table 3:** Summary of the biochemical parameters in children with a congenital solitary functioning kidney according to estimated glomerular filtration rate.

	eGFR		
	<90 mL/min/1.73 m^2^	>90 mL/min/1.73 m^2^	*p*
	*n* = 13	*n* = 55	
Urea (mg/dL)	19.3 (7.25–42.9)	28.0 (25.0–34.0)	0.213
Uric acid (mg/dL)	5.2 (7.25–42.9)	4.6 (4.0–4.9)	0.359
Galectin-3 (pg/mL)	378.9 (284.2–444.9)	270.9 (163.5–362.2)	0.049
Cystatin C (mg/L)	1.13 (0.98–1.88)	0.94 (0.77–1.14)	0.012

Data are given as the median (Me) with interquartile range (IQR).
